# Digestive Manifestations of Post-COVID-19: A Focus on Therapeutic Strategies

**DOI:** 10.3390/pathogens14060555

**Published:** 2025-06-03

**Authors:** Cristina Stasi, Massimo Bellini

**Affiliations:** 1 Department of Life Science, Health and Health Professions, Link Campus University, 00165 Rome, Italy; 2 Gastrointestinal Unit and Regional Center for Functional and Motility Digestive Disorders, Department of Translational Research and New Technologies in Medicine and Surgery, University of Pisa, 56124 Pisa, Italy; massimo.bellini@unipi.it

**Keywords:** SARS-CoV-2, COVID-19, post-COVID-19, digestive manifestations, irritable bowel syndrome, antiviral agents, therapeutic strategies, probiotics

## Abstract

Post-COVID-19 is a chronic infection-related syndrome, including exacerbations of pre-existing or newly diagnosed conditions that have been established after the acute phase of COVID-19 and have demonstrated a wide range of systemic effects beyond the lungs. SARS-CoV-2 attaches to its receptor, angiotensin-converting enzyme 2 (ACE-2). Transmembrane serine protease 2 (TMPRSS2) facilitates viral entry and spread. ACE-2 receptors are detectable in several tissues, including the respiratory mucosa, digestive tract, heart, kidney, and brain. Several investigations have demonstrated an increase in digestive manifestations post-acute COVID-19, likely related to an alteration in the intestinal microbiota following infection. These changes can lead to a loss of species diversity, resulting in an overgrowth of opportunistic pathogens and deprivation of commensal bacteria. In this context, post-infection irritable bowel syndrome shows an increased incidence compared to controls. Growing evidence also suggests the enduring presence of SARS-CoV-2 in the gut tissue. Studies are ongoing to investigate antiviral agents that counteract prolonged COVID-19 symptoms. Therefore, the objectives of this review were to summarize the digestive manifestations, focusing on irritable bowel syndrome and therapeutic strategies. This review gives an overview of studies published in English in the last two years on the PubMed database.

## 1. Introduction

The World Health Organization defined post-COVID-19 as a syndrome characterized by a range of symptoms, which usually start within 3 months of the initial COVID-19 illness and last at least 2 months [1]. Therefore, post-COVID-19 is an infection-associated chronic condition, including exacerbations of preexisting or new-onset conditions, which develops after the acute phase of COVID-19, and demonstrates a broad range of systemic effects beyond the lungs [2]. Many of the studies were conducted 2 years after the pandemic. A recent systematic review and meta-analysis [3] highlights that COVID-19 survivors continue to experience symptoms and functional impairment even more than one year after the initial infection.

Globally, Al-Oraibi et al. [4] estimated the pooled prevalence and analyzed the most common post-COVID-19 symptoms among 6481 healthcare workers infected with acute respiratory syndrome coronavirus 2 (SARS-CoV-2). The systematic review and meta-analysis found a pooled prevalence of long COVID-19 of 40%, with a median monitoring period of 22 weeks. The most frequent symptoms included fatigue (35%), neurological symptoms (25%), loss/decrease in smell and/or taste (25%), myalgia (22%), and shortness of breath (19%). Accordingly, a previous meta-analysis by Luo et al. [5] investigated the prevalence and determinants of prolonged symptoms after COVID-19, examining 211 studies with a total of 13,368,074 individuals. Within the first 3 months post-infection, the most reported symptoms were fatigue, post-traumatic stress disorder, mood disorders, and dyspnea. At 12 months and beyond, fatigue, dyspnea, psychiatric/neurological disorders, and joint pain were the frequently occurring manifestations. Determinants for prolonged symptoms included being a female, older, having severe COVID-19 during the acute phase, multiple medical conditions, longer hospital stays, and a higher basic metabolic rate.

SARS-CoV-2 attaches to its receptor ACE-2 (angiotensin-converting enzyme 2), and transmembrane serine protease 2 (TMPRSS2) promotes viral entry and spread [6]. The ACE-2 receptors are detected in the respiratory mucosa, gastrointestinal (GI) tract, heart, kidney, and brain [7]. The digestive manifestations may persist or newly develop following the acute phase of COVID-19 [8,9], affecting patients’ quality of life and complicating recovery.

Psychological disorders frequently occur after viral infections. The COVID-19 pandemic triggered a 25% increase in the prevalence of anxiety and depression worldwide [10]. Sperber et al. [11] reported a worldwide prevalence of irritable bowel syndrome (IBS) based on Roma IV criteria of 4.1% (range: 3.9–4.2%). In this context, the intestinal microbiota, which can produce 5-HT and is also modulated by 5-HT, could be crucial in the development of IBS and associated mood disorders through the microbiota–gut–brain axis [12].

Based on these premises, the objectives of this review were to summarize the digestive manifestations, with a particular focus on IBS and therapeutic strategies for the post-COVID-19 syndrome. To reach this objective, we conducted a literature search using the PubMed database for relevant studies published in English, particularly in the last two years.

## 2. Post-COVID-19 Digestive Manifestations

The alteration in the gut microbiota following COVID-19 has been the subject of close attention, and it can be hypothesized that the enduring dysbiosis following recovery may be related to long COVID-19 syndrome [13].

Li et al. [14] studied the gut microbiome profile among 13 asymptomatic infections, 24 post-acute COVID-19 syndrome patients, 31 discharged patients with SARS-CoV-2 re-positive, and 13 non-COVID-19 healthy controls. All the patients were unvaccinated. The asymptomatic and symptomatic patients presented different gut microbiome profiles. In particular, the microbiome profile of asymptomatic infected patients was enriched with short-chain fatty acids (SCFAs)-producing species *Faecalibacterium prausnitzii*, *Blautia obeum*, *Roseburia hominis*, and *Gemmiger formicilis*. Moreover, the microbiome profile of asymptomatic infected patients and patients who tested re-positive after discharge presented other SCFA-producing species, *Bifidobacterium longum*, *Eubacterium ramulus*, *Phascolarctobacterium faecium*, *Butyricicoccus pullicaecorum*, and *Lactobacillus amylovorus*; these were also inversely correlated with poor prognosis in COVID-19 cases.

Table 1 summarizes key differences in gut microbiota profiles between post-COVID-19 patients and controls.

Marasco et al. [19] conducted a multicenter study including 871 hospitalized patients (575 with COVID-19 and 296 without) to assess the prevalence of digestive symptoms. They found that nausea and diarrhea were more common in the COVID-19 group (59.7% of patients) compared to the non-COVID-19 group (43.2%). A month after admission, some patients continued to experience nausea. Accordingly, Ashktorab et al. [20], in 39 patients experiencing digestive symptoms, of whom 61.5% had nausea and vomiting, observed that patients experiencing vomiting during the acute infection were more likely to suffer from digestive manifestations associated with post-COVID-19.

A subsequent systematic review was conducted by Hawkings et al. [21] from early 2020 to mid-2023 in 28 different countries and included 45 studies, for 2,224,790 patients. An overall weighted prevalence of 10.8% of persistent digestive symptoms of any nature was found compared with 4.9% in healthy controls. Persistent symptoms of any duration showed a prevalence ranging from 0.2% to 24.1% in seven studies with a median monitoring period of 18 weeks. The incidence was evaluated from early 2022 to 2023, showing a high incidence of functional gastrointestinal disorders (FGIDs) after recovery from acute COVID-19, which highlighted an association between previous SARS-CoV-2 exposure and the occurrence of functional digestive disorders. Among the most incident frequent symptoms of post-acute COVID-19 across healthcare settings in seven countries [22], joint pain (from 1.6 to 14.3), abdominal pain (from 0.3 to 9.9), and other digestive manifestations (from 0.6 to 13.3), cough (from 0.3 to 9.1), and anxiety (from 0.8 to 11.4) were reported.

Baalbaki et al. [23] conducted a systematic literature review to evaluate whether omics-based analyses of different symptom-based phenotypes can contribute to understanding pathophysiological mechanisms and identify potential biomarkers and relevant traits in long-term COVID-19 patients compared to recovered individuals or healthy controls. In particular, studies on the microbiome using the technique of shotgun metagenomics have highlighted a relationship between altered microbiome composition, metabolic modifications, and the spectrum of neurological symptoms presented as a post-COVID-19 syndrome. Mohammed et al. [24] provided a systematic overview of 24 studies of digestive manifestations, excluding hepatic ones, and clinical implications in COVID-19 patients with abdominal symptoms, categorizing them into GI infections and inflammations, vascular disorders, structural abnormalities, other diagnosed abnormalities, and undiagnosed conditions. In the GI infections group, infections from *Cryptosporidium* species and *Helicobacter pylori* up to mucormycosis were reported, likely as a consequence of immunodeficiency or imbalance in microbiota composition. Among undiagnosed post-COVID-19 GI conditions, weight loss and FGID-like symptoms have been observed, highlighting the need for further research to understand persistent chronic manifestations after COVID-19 infection. Among inflammatory conditions, ulcerative colitis and acute pancreatitis have been reported in more severe cases, highlighting the impact of severe COVID-19 on the GI system and the need to monitor these types of patients recovering from COVID-19. Accordingly, a most recent study [25] conducted on 90 Inflammatory Bowel Disease (IBD) patients with a non-severe form of COVID-19, of whom 88.9% received at least standard two doses of vaccine against COVID-19, demonstrated that 19.30% of patients presented exacerbated digestive manifestations during COVID-19 and 38.1% of these developed post-COVID-19.

Choudhury et al. [26] analyzed the specific post-COVID-19 phenotype with digestive manifestations. In this systematic review and meta-analysis of studies reporting digestive manifestations in 12% of patients after COVID-19 and 22% as part of post-COVID syndrome, the most common symptoms include the following:

- Loss of appetite
- Loss of taste
- Abdominal pain
- Diarrhea
- Nausea/vomiting

The reported pooled rate of IBS after COVID-19 was 0.17 (95% CI, 0.06–0.37, I2 = 96%) [26].

Regarding patients with FGID, Marasco et al. [27] in a meta-analysis highlighted that COVID-19 survivors were at risk of new onset of IBS compared to controls. A more recent post hoc analysis of a prospective multicenter cohort study [28] compared the psychological burden in patients with post-COVID-19 gut–brain interaction disorders with those with pre-existing IBS/functional dyspepsia (FD) and controls without gut–brain interaction disorders. Among patients with post-COVID-19 gut–brain interaction disorders, FD was the most prevalent. Irritable bowel syndrome was reported in 37.0% of patients. Furthermore, the study underlined that patients with post-COVID-19 gut–brain interaction disorders experienced a significant worsening.

All studies conducted so far underline the need to monitor patients for exacerbation of pre-existing gastroenterological conditions and evaluate a possible new onset in post-COVID-19 patients.

This review focuses specifically on IBS, as a high incidence of IBS has been reported among new-onset FGIDs in many studies, in addition to the significant impact on the quality of life of patients affected by this disorder. Furthermore, post-infectious (PI)-IBS can develop following acute infectious gastroenteritis. Given that gastrointestinal manifestations are commonly reported during the acute phase of COVID-19, a possible contributing factor to the increased incidence of IBS could be COVID-19-related gastroenteritis.

## 3. Post-COVID-19 Irritable Bowel Syndrome

Several pathophysiological mechanisms are implicated in the onset of IBS post-COVID-19. Pieces of evidence suggest that the microbiota profile plays a potentially more proactive role in the gut microbiome, where pre-existing or early-infection microbial profiles are involved in both the severity of COVID-19 and the development of post-COVID-19 sequelae [14]. The intestinal microbiota can affect motility and visceral perception via the brain–gut axis and the key role of integrative brain structures. Moreover, serotonin [12,29], neuropeptide Y [29,30], dopamine, γ-aminobutyric acid, and histamine [31] could be crucial in the modulation of gut–brain interactions [29,30]. The mechanism behind post-COVID-19 IBS resembles that of post-infectious IBS. An abnormal microbiota–gut-brain axis can lead to a dysregulation of intestinal neurotransmitters [31], resulting in visceral hypersensitivity and altered GI motility [32].

SARS-CoV-2 invades the intestine through ACE-2, which is highly present in the absorptive enterocytes of the ileum and large intestine, and through the TMPRSS2 receptor, which cleaves the coronavirus spike protein in the cell membrane, releases the fusion peptide into the membrane [33].

Edwinson et al. [34], for the first time, studied the role played by human microbiota in regulating the expression of ACE-2 receptors in the GI tract. They enrolled 12 patients (11 females) with Rome III IBS and 6 healthy volunteers who underwent sigmoid colon biopsies and pooled fecal samples for shotgun metagenomics to assess microbiota composition. IBS patients showed reduced microbial α-diversity and changes in microbiota composition. In particular, the phylum *Methanobacteriota*, the families *Odoribacteraceae*, *Methanobacteriaceae*, *Odoribacteraceae*, and *Sutterellaceae*, and the genus *Methanobrevibacter* decreased in IBS patients. The phylum *Actinomycetota* was enhanced in IBS patients compared with healthy controls. However, the study demonstrated a similar ACE-2 detection in colonic biopsies from IBS patients and healthy subjects. In the same study, a humanized mouse model demonstrated that healthy commensal microbiota and altered microbiota from IBS patients inhibited ACE-2 expression, suggesting the key role of commensal microbiota in regulating the expression of ACE-2 in the colon.

Apart from the involvement of ACE-2 receptors in the renin–angiotensin system, these receptors have a key function in the intestinal uptake of dietary amino acids, such as tryptophan [35], which regulates, via mTOR pathway activation, the secretion of antimicrobial peptides. These influence the composition of the intestinal microbiota and the susceptibility to inflammation of the large intestine [33]. Wong et al. [36], in patients experiencing persistent symptoms (3–22 months after acute infection) compared with 60 patients with acute COVID-19 and 30 individuals with symptom-free recovery from COVID-19, demonstrated a viral inflammation-driven serotonin depletion linked to a reduction in tryptophan absorption, thrombocytopenia, and increased MAO expression. The downregulation of ACE-2 receptors during the acute phase of COVID-19 in target cells contributes to SARS-CoV-2 pathogenesis, upregulating angiotensin II, which modulates the gene expression of several inflammatory cytokines via NF-κB signaling [37]. Cytokines and an altered microbial composition can induce systemic modulation of the brain structures and functions through the gut microbiota–immune system–brain axis [38,39].

**Figure 1:**
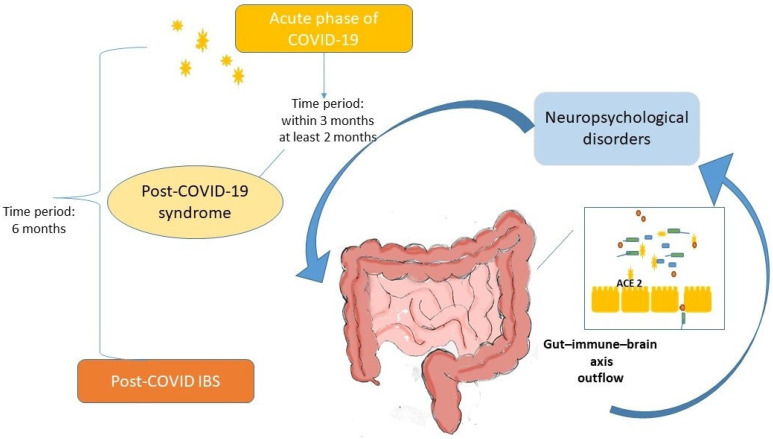
Proposed pathophysiological mechanisms of post-COVID-19 irritable bowel syndrome. Legend: SARS-CoV-2 binds to ACE-2 receptors present in enterocytes of the ileum and large intestine, influencing the function of intestinal absorption of dietary amino acids, such as tryptophan, and the secretion of antimicrobial peptides. In the intestine, the virus alters the composition of the microbiota. Downregulation of receptors increases the gene expression of several inflammatory cytokines that induce epithelial and barrier damage. Viral persistence and immune and microbiota alterations can affect motility and visceral perception by neurotransmitters, leading to a systemic alteration in brain functions through the gut microbiota–immune–brain axis, resulting in post-COVID-19 irritable bowel syndrome-like symptoms among post-COVID-19 syndrome (within 3 months), confirming post-COVID-19 irritable bowel syndrome with a minimum of 6 months of symptom onset.

Figure 1 summarizes the possible pathophysiological mechanisms underlying post-COVID-19 IBS.

A Chinese study [15] developed a large single-site dataset covering different diseases, using a machine learning multi-class model to identify their fecal microbiome profiling. In this large cohort, the presence of *Klebsiella pneumoniae*, a well-characterized opportunistic pathogen, showed a positive association with Crohn’s disease, colorectal cancer, IBS-D, obesity, post-acute COVID-19 syndrome, and ulcerative colitis. The dysbiotic microbiome in post-COVID patients is characterised by a decrease in beneficial SCFA-producing bacteria and an increase in opportunistic bacteria [40].

Noviello et al. [41] demonstrated that loose stools, chronic fatigue, and somatization were increased 5 months after SARS-CoV-2 infection in comparison with control subjects. Ghoshal et al. [42], in a case–control study, using translated validated Rome Questionnaires, found that patients with previous COVID-19 had a higher probability of developing chronic bowel habit changes, dyspeptic symptoms, and their overlap during the first 3-month monitoring period and IBS (particularly IBS-D), uninvestigated dyspepsia (UD), and IBS–UD overlap during a 6-month monitoring period compared with the healthy controls. Moreover, the presence of digestive manifestations during COVID-19 was correlated with a higher occurrence of new onset of FGIDs in the follow-up. Accordingly, Marasco et al. [43] found that COVID-19 patients reported higher rates of IBS (3.2%), according to Rome IV criteria, than the control group (0.5%) at 12 months of follow-up. Table 2 summarizes the studies reporting post-COVID-19 IBS.

A single UK center case series [44] demonstrated the onset of chronic digestive symptoms, suggesting new post-COVID-19 digestive symptoms for 43.8% of the subjects involved in the study, 6 months after the acute phase of COVID-19. Accordingly to this suggestion, Austof et al. [45] studied the association between the occurrence of digestive symptoms during the acute phase and the development of post-COVID IBS according to Rome IV criteria in a cohort of 1475 subjects, of whom 33.8% developed digestive symptoms. The finding showed that patients with digestive symptoms during the acute phase of COVID-19 were slightly younger, more likely female, more prone to perceive stress, and with a more severe acute form than those without digestive symptoms. The incidence of post-COVID-19 reached 3.0%.

A Polish prospective, single-center study [46] evaluated, among 257 patients, the occurrence of digestive symptoms through the Rome IV Diagnostic Questionnaire for FGID in adults immediately after discharge and at 3 and 6 months post-COVID-19. In this study, the IBS-like symptoms were present after 3 months in 14 patients (5.4%), and IBS criteria were satisfied after 6 months in 15 patients (5.8%). A Turkish cross-sectional study [47] conducted from November 2020 to February 2021 on 233 eligible patients revealed a prevalence of 11.6% of IBS based on Rome IV criteria, 27.4% of depression, and 36.9% of anxiety assessed by the Hospital Anxiety and Depression Scale after COVID-19. Siyal et al. [48] evaluated the incidence of post-COVID-19 IBS based on Rome-IV criteria in 178 COVID-19 patients after discharge. IBS was found in 10.6% of patients, of whom 53.1% presented diarrhea-predominant, 31.2% constipation-predominant, and 15.6% mixed-type IBS. Risk factors were female sex, oxygen therapy, and high procalcitonin levels during the acute infection. A prospective study [49] highlighted a significantly higher number of new-onset disorders of gut–brain interaction compared with healthy controls at 3 and 6 months of the monitoring period. Accordingly, Zhang et al. [50] evaluated the incidence of post-COVID-19 FGID in 190 Chinese COVID-19 patients, finding an increment of new-onset post-COVID-19 FGID at 6 months compared with healthy controls, suggesting that digestive symptoms during the acute phase were an independent risk factor.

## 4. Therapeutic Strategies

Currently, the treatment for IBS patients follows the strategies provided by Italian guidelines [51]; however, some evidence suggests specific approaches, such as dietary interventions, probiotics, and antivirals.

COVID-19 causes changes in the gut microbiota, including a loss of species diversity in the gut, an overgrowth of opportunistic pathogens, and a deprivation of commensal bacteria. These changes may also affect the expression of ACE-2 receptors, which may influence the severity of COVID-19. Therefore, the therapeutic strategy for post-COVID-19 includes several targets, many of which are directed at modulating the intestinal microbiota, especially when symptoms are primarily GI.

## 5. Dietary, Prebiotics, Probiotics, Postbiotics, and Other Therapeutic Approaches

A single-center, double-blinded, and placebo-controlled randomized (1:1) trial was conducted by Calvani et al. [52] to investigate the effects of beetroot juice supplementation (200 mL) on physical function, gut microbiota, and inflammatory state in 31 adult patients affected by long COVID-19. The 14-day supplementation demonstrated changes in gut microbiota and inflammatory markers, as well as in fatigue resistance and the distance covered on the 6 min walk test, which increased from baseline. The microbiota significantly shifted toward bacteria with beneficial effects, such as *Akkermansia*, *Oscillospira*, *Prevotella*, *Roseburia*, *Oscillospiraceae*, and *Turicibacter*, in patients receiving beetroot juice supplementation compared with placebo.

A secondary analysis of this randomized controlled clinical trial by Marzetti et al. [53] investigated the effect of beetroot juice administration on circulating markers of mitochondrial quality and inflammatory markers and their relationship in 15 adult participants with long COVID-19 compared to 10 assigned to placebo. The study demonstrated that beetroot juice administration for 14 days reduced IL-1β, IL-8, and tumor necrosis factor-alpha serum levels. Still, mitochondrial quality markers did not show differences between the placebo and beetroot juice groups. An inverse correlation was found between vesicular markers of mitochondrial quality and performance on the 6 min walk test and flow-mediated dilation, regardless of group assignment, suggesting improved physical performance and endothelial function.

Ribeiro et al. [54] studied the association between food consumption and the onset of post-COVID syndrome in 1322 elderly Brazilians. They found that fruit consumption was associated with a lower incidence of this syndrome, while consumption of sugary drinks, cookies, sweets, and snacks was associated with a higher incidence. Accordingly, Barghchi et al. [55], in 246 recovered COVID-19 patients, found a relationship between good-quality food intake and psychological disorders during the post-infection period. Fruits, legumes, nuts, and whole grains were associated with a reduced risk.

Further insights will be available with the results of an ongoing trial involving the consumption of medical food, KetoCitra^®^ [56], which provides exogenous beta-hydroxybutyrate to increase blood beta-hydroxybutyrate levels, inducing inflammatory inhibition and anti-inflammatory effects without a strict ketogenic diet.

Several lines of evidence suggest that prebiotics and probiotics contribute to mitigating neuroinflammation, with subsequent improvement of neuro-psychological state. Prebiotics, probiotics, and gut microbiota contribute to the formation of metabolites, including the neurotransmitters GABA and serotonin, and are crucial for maintaining psychological well-being. Alterations in their levels contribute to stress-related disorders. Given that digestive symptoms in post-COVID-19 IBS can be linked to viral persistence, gut microbiome, and immune alterations, affecting the gut–brain axis, evidence suggests the therapeutic effects of prebiotics and probiotics [57].

Giacosa et al. [58] explored the anti-inflammatory and intestinal antimicrobial modulating effects exerted by 30-day supplementation of Boswellia and Curcuma extracts combined with a low-FODMAP diet in 16 patients with long COVID-19 and IBS-like symptoms and 28 subjects with IBS without previous COVID-19 infection. Patients were recruited 60–120 days after the end of COVID-19 infection. The findings showed that both cohorts had a statistically significant decrease in bloating and relief of abdominal pain. However, enteral dysbiosis significantly decreased only in IBS patients, suggesting that patients with long COVID-19 and IBS-like symptoms may benefit from etiology-specific strategies for dysbiosis.

Horvath et al. [59] investigated the gut–lung axis to explore the effects of a probiotic in post-acute COVID-19 disease by a randomized, placebo-controlled trial to test the effects of a probiotic (Pro-Vi 5) for 6 months in 21 patients with a mild form of COVID-19. They found an improvement in tiredness, psychological state, microbiome composition, microbial-derived metabolites, lipoprotein levels, and markers of innate and adaptive immunity compared to controls. These findings suggest the treatment of post-acute COVID-19 syndrome during the monitoring period of 3–6 months.

Lau et al. [60] assessed a symbiotic (prebiotics and probiotics) preparation (SIM01) for the improvement of post-COVID-19 syndrome by a randomized, double-blinded, placebo-controlled trial in 463 patients with 14 post-COVID-19 symptoms, including digestive ones. At 6 months, significantly higher proportions of the SIM01 group had alleviation of fatigue, memory loss, difficulty in concentration, digestive symptoms, and general unwellness compared with the placebo group.

Ranisavljev et al. [61] conducted a randomized (1:1), placebo-controlled, double-blinded design clinical trial in people aged 18–65 years affected by post-COVID-19 syndrome, investigating the role played by the supplementation synbiotic mixture including *Lactobacillus rhamnosus DSM32550*, Humiome^®^ *Lactiplantibacillus plantarum DSM 34532*, *Bifidobacterium animalis* subsp. *lactis DSM 32269*, *Bifidobacterium longum DSM 32946*, fructooligosaccharides, and zinc over 3 months. Findings demonstrated an amelioration of tissue metabolism and clinical features of post-COVID-19 fatigue syndrome.

The use of probiotics could be suggested for their potential multiple actions, including restoring intestinal microbiota, modulation of GI motility, visceral hypersensitivity, pain, and mucosal immune activation, which in turn improved gut–brain bidirectional interactions [51].

Postbiotics are diverse compounds, including bacteriocins, complex proteins, hydrogen peroxide, organic acids, exopolysaccharides, enzymes [62], SCFA, vitamins, and tryptophan [63], that have demonstrated immunomodulatory, anti-inflammatory, and antiviral effects. A wide range of postbiotics was secreted by probiotics with several beneficial effects on mental health.

With the premise that the anti-inflammatory effect of vitamins K2 and D3 attenuate the course of acute COVID-19 infection, Atieh et al. [64] investigated the role played by vitamins K2/D3 administration on long COVID-19 symptoms, gut and inflammatory markers in 151 long COVID-19 patients, demonstrating a significant improvement in the RECOVER Long COVID Index, the number of long COVID-19 symptoms, and several gut and inflammatory markers, such as markers of monocyte activation, high-sensitivity C-reactive protein, IL-6, inducible protein of 10 kDa, tumor necrosis factor receptors 1 and 2, and intercellular adhesion molecule-1. Accordingly, the supplementation with Pro Resolving lipid mediators (composed of 17-HAD, 14-HAD, and 18-HEPE) combined with telerehabilitation in ARACOV, a multicenter study, has been demonstrated to reduce the symptoms of patients with post-COVID-19.

Considering post-COVID-19 syndrome, as a consequence of alterations in the gut microbiome, Lau et al. [65] conducted a prospective, nonrandomized, open-label, interventional study, demonstrating that fecal microbiota transplantation improves insomnia and anxiety post-COVID-19-related by promoting the restoration of a “normal” gut microbiome.

## 6. Antiviral Approach

Growing pieces of evidence suggest the persistence of SARS-CoV-2 in the gut. Peluso et al. [66] by whole-body positron emission tomography (PET) using the radiopharmaceutical agent to trace activated T lymphocytes in 24 subjects at different time points, ranging from 27 to 910 days after the acute phase of COVID-19. The study provides evidence of persistent T-cell activation in a variety of tissues, including intestinal tissue, suggesting potential ongoing viral transcriptional activity, resulting in an active viral reservoir. Thus, even a clinically mild infection could have long-term consequences. Zollner et al. [67] demonstrated viral persistence in the gut mucosa about 7 months after mild acute COVID-19 in 32 of 46 patients with IBD who underwent endoscopy. In a large cohort of 381 subjects, Ghafari et al. [68] demonstrated viral persistence for at least 30 days. In 54 patients, viral persistence was also demonstrated for at least 60 days. Persistent infection had more than 50% higher odds of self-reporting long COVID-19 compared with patients without persistent infection. Zuo et al. [69], in a single-center, cross-sectional cohort study, evaluated the virus persistence at different time points following recovery from mild COVID-19 and its association with long COVID-19 symptoms in 225 patients (201 residual surgical specimens, 59 bioptic samples taken during GI endoscopy, and 57 blood component samples). Viral RNA was detected in different tissues, including the liver, kidney, stomach, intestine, brain, blood vessel, lung, breast, skin, and thyroid, demonstrating an association between viral persistence and long COVID-19 symptoms.

Based on these premises, several antiviral agents were used to counteract long COVID-19 symptoms.

Gostimirovic et al. [70] summarized the effects of resveratrol in post-COVID-19-related disorders. Resveratrol exhibits antiviral activity as it can act through inhibition of viral replication, protein synthesis, and inhibition of transcription and signaling pathways [71]. Most of the metabolites derived from resveratrol reach the lower GI tract, where they interact with the intestinal microbiota, particularly in diseases associated with gut dysbiosis, such as inflammatory bowel diseases and obesity [70].

Iannou et al. [72] investigated the effectiveness of outpatient treatment of COVID-19 with nirmatrelvir–ritonavir in preventing post-COVID-19 syndrome in the Veterans Health Administration. However, in 31 potential post-COVID-19 patients, only thromboembolic events seemed to be reduced by this treatment. A most recent phase 2 clinical trial conducted by Krumholz et al. [73] investigated the efficacy and safety of 15 days of nirmatrelvir/ritonavir (Paxlovid, ClinicalTrials.gov ID: NCT05668091) [74] compared with placebo/ritonavir in 100 highly symptomatic adult participants with long COVID-19. Although this study (ClinicalTrials.gov ID: NCT05668091) [75] did not show a significant benefit for a 15-day regimen of nirmatrelvir–ritonavir for long-term COVID-19 symptoms, the findings suggest further investigation with longer treatment regimens, other antiviral agents or combinations of these, and interventions targeting multiple post-COVID pathological mechanisms.

The pathophysiology of long COVID-19 is quite complex, with various clinical characteristics potentially linked to different pathways involved in the onset of this syndrome. As a result, the potential pharmacological targets may also differ. Several studies have demonstrated the persistence of viral RNA and/or viral particles [66,67].

In some patients with chronic hepatitis C virus infection, extrahepatic manifestations may persist even after the virus has been cleared after treatment [76,77]. However, patients who achieve a sustained virologic response to treatment with direct antiviral agents are at a lower risk of extrahepatic manifestations compared to those who do not respond to treatment [77]. Similarly, COVID-19-related conditions may be associated with an inflammatory state that triggers dysregulation of the immune system, gut microbiota, and gut–brain axis, which may remain altered in some patients even after the virus is eradicated.

Several pieces of evidence [78] highlighted that most existing therapeutic strategies target IBS, which is not associated with COVID-19.

Specifically, the treatment of IBS is based on the recommendations of guidelines [51] and depends on the prevalent symptomatology and its etiology. Therefore, here, we review the key studies on the strategies, specifically, implemented for patients with post-COVID-19 syndrome, including those with post-COVID-19 IBS. The treatment considered focuses on the potential antiviral effects of compounds that do not directly target the virus but instead modulate intestinal microbiota.

We have also considered emerging antiviral strategies, with particular attention to ongoing trials, which may further change therapeutic options for post-COVID-19 manifestations.

Therefore, while probably not all patients may be suitable candidates for antiviral therapy, those who are candidates due to the ongoing presence of the virus should be carefully monitored not only for clinical symptoms but also undergo tests to assess the persistence of the virus or confirm its eradication. Zuo et al. [69] detected SARS-CoV-2 RNA in various tissues (at 1 month, 2 months, and 4 months after infection) and observed an association between viral persistence in the body and post-COVID-19 symptoms. In some cases, the persistence of the virus may require a longer treatment period or a combination of drugs.

Several trials are evaluating the safety and efficacy of antiviral agents, including Truvada or Selzentry (ClinicalTrials.gov ID: NCT06511063) [79], Paxlovid (ClinicalTrials.gov ID: NCT05595369; ClinicalTrials.gov ID: NCT05965726, ClinicalTrials.gov ID: NCT06441955, ClinicalTrials.gov ID: NCT05576662, ClinicalTrials.gov ID: NCT05823896, ClinicalTrials.gov ID: NCT05852873) [80,81,82,83,84,85], antiviral and inflammation-controlling LAU-7b (ClinicalTrials.gov ID: NCT05999435) [86], Ensitrelvir (ClinicalTrials.gov ID: NCT06161688) [87], Remdesivir (ClinicalTrials.gov ID: NCT04978259, ClinicalTrials.gov ID: NCT05911906) [88,89] Valacyclovir Plus Celecoxib (ClinicalTrials.gov ID: NCT06316843) [90], and Paxlovid/remdesivir (ClinicalTrials.gov ID: NCT06792214) [91], most of which are active and therefore without publication of the results.

The randomized clinical study by Geng et al. [92] (ClinicalTrials.gov Identifier: NCT05576662) [83] demonstrated that a 15-day course of Nirmatrelvir/Ritonavir in a population of patients with PASC, although found to be safe, did not demonstrate a significant benefit in improving symptoms in a cohort of vaccinated patients with post-COVID-19. Table 3 summarizes the trials on antiviral treatments in post-COVID-19. Long-term follow-up results from a randomized trial [93] (ClinicalTrials.gov ID: NCT04978259), evaluating the effects of remdesivir on recovery one year after hospitalization for COVID-19 in 208 patients, reported no significant differences between remdesivir and standard of care in quality of life or symptoms.

Alternative to direct-acting antivirals, Berbamine dihydrochloride can limit the damage produced to intestinal cells through an autophagy mechanism [94].

## 7. Conclusions

Digestive symptoms are one of the main manifestations of post-COVID-19. To date, several studies conducted on large cohorts underscore the need for monitoring patients for exacerbation of pre-existing gastroenterological conditions and evaluating the new onset of brain–gut axis disorders in post-COVID-19 patients. Most studies conducted in various countries using the Rome IV criteria suggest that the incidence of post-COVID-19 IBS varies widely, ranging from 1.1 to 11.6%. Given its impact on quality of life, early recognition and a multidisciplinary approach are crucial for the management of post-COVID-19 IBS patients. Currently, several clinical trials are being conducted to test antiviral drugs that can act on the virus reservoir at any level where it is located. Some evidence suggests that these manifestations are due to the virus’s persistence in the GI tract. The results of the trials conducted so far do not appear to demonstrate a significant improvement in symptoms after antiviral treatment. However, these findings suggest that it is essential to await the results of studies conducted with longer-term antiviral treatment, or other antiviral agents, or a combination of these. The ongoing research results are crucial for understanding pathogenesis and targeted therapies. Emerging studies support the role of microbiome-targeted therapies.

Therefore, while the treatment of patients with IBS continues to follow the strategies provided by international guidelines, longitudinal studies are needed to thoroughly investigate the evolution of post-COVID-19 manifestations, especially for those manifestations, such as IBS, which have shown a higher incidence in the studies conducted so far, and the burden of long COVID-19 appears to be growing.

## Figures and Tables

**Table 1:** Microbiota profile in post-COVID-19 syndrome.

Authors	Study Design	Country	Study Population	Microbiota Profile in Controls	Microbiota Profile in Post-COVID-19 Syndrome
Li et al. [14]	Longitudinal study	China	13 asymptomatic infections, 24 post-acute COVID-19 syndrome patients, 31 discharged patients with SARS-CoV-2 re-positive, and 13 non-COVID-19 healthy controls	Asymptomatic infected patients and patients without symptoms after discharge had significantly higher microbial diversity than patients with adverse outcomes. A relative abundance of *Bacteroides* was found in non-COVID-19 healthy subjects	Post-acute COVID-19 syndrome patients were enriched with opportunistic pathogens (*Escherichia coli*, *Clostridium ramosum*, *Klebsiella ornithinolytica*, and *Hungatella hathewayi*)
Su et al. [15]	Machine-learning methods	China	2320 individuals with different characterized phenotypes (174 colorectal cancer; 168 colorectal adenomas; 200 Crohn’s disease; 147 ulcerative colitis; 145 irritable bowel syndrome—diarrhea subtype; 148 obesity; 143 cardiovascular disease; 302 post-acute COVID-19 syndrome and 893 healthy controls	Compared to controls, almost all disease states were associated with a decreased abundance of *Bacillota* or *Actinomycetota* and an increase in *Bacteroidota*	Post-acute COVID-19 syndrome and other different phenotypes (Crohn’s disease, colorectal cancer, irritable bowel syndrome—diarrhea subtype, obesity, ulcerative colitis) were positively associated with *Klebsiella pneumonia* and negatively correlated with *Roseburia intestinalis* Post-acute COVID-19 syndrome showed a significant increase in abundance of *Phocaeicola vulgatus* and *Bacteroides xylanisolvens*, while those with UC were enriched in *Bacteroides ovatus*
Comba et al. [16]	Prospective	US	799 subjects: 380 positive and 419 negative for SARS-CoV-2. Within the 1-year follow-up, 80 positive patients for SARS-CoV-2 developed long COVID-19	SARS-CoV-2-negative subjects had higher α-diversity based on the Chao1 metric but comparable based on the Shannon index	The presence of some specific species during the acute phase, such as *Prevotella* species, *Leuconostoc* species, and members of the *Lactobacillaceae* family like *Eubacterium* species and *Agathobacter* species predict long COVID-19 Changes in *Lachnospiraceae* were associated with the development of digestive symptoms
Ferreira-Junior et al. [17]	Longitudinal study	Brazil	149 patients, months after having acute COVID-19, of whom approximately 39% developed clinical manifestations after the acute phase; 71 controls	Compared with controls, differences in the microbiota diversity in post-COVID-19 patients	Possible association between post-COVID-19 dysbiosis and some genera, including *Desulfovibrio, Haemophilus, Dialister,* and *Prevotella*, in addition to decreased beneficial microbes, associated with antibiotic-induced dysbiosis, such as *Bifidobacterium* and *Akkermansia*
Su et al. [18]	Cross-sectional and longitudinal cohorts	China	1207 with post-COVID-19 (n = 1011 in two cross-sectional cohorts and n = 196 longitudinal cohort) A cohort of 201 previous COVID-19 subjects without post-COVID-19 syndrome and a cohort of 653 healthy subjects without COVID-19 exposure were employed as non-Post-COVID-19 controls	The diversity (Shannon) and richness (observed number of species) of the gut microbiome in the post-COVID-19 syndrome were significantly lower than control group	Enrichment of opportunistic pathogens, such as *Klebsiella quasipneumoniae* and *Mediterraneibacter gnavus*, in subjects with post-COVID-19 syndrome *Coprobacillus cateniformis* being positively associated with most digestive symptoms

**Table 2:** Studies reporting post-COVID-19 irritable bowel syndrome.

Authors	Study Design	Country	Study Population	Methods	Time Interval Between COVID-19 and Post-COVID-19 IBS
Ghoshal et al. [42]	Case–control study	India Bangladesh	280 COVID-19 patients and 264 controls	Follow up at 1, 3, and 6 months using translated validated Rome Questionnaires	At 6 months 15 (5.3%) developed IBS and 5 (1.8%) IBS–UD overlap
Marasco et al. [43]	Prospective, multicenter, controlled study	Italy	883 hospitalized patients without digestive symptoms (614 COVID-19 patients and 269 controls)	Follow up at 1, 6, and 12 months post-hospitalization by Rome IV criteria	At 6 months 0.5% of COVID-19 patients developed IBS versus 3.2% in controls
Cooney et al. [44]	Single-center case series	UK	122 COVID-19 patients, of whom 48 completed the follow-up survey	Weblink to a symptom survey at the point of their acute COVID-19 illness, and 6 months later, a follow-up survey	At 6 months new digestive symptoms affecting 21 patients (43.8 %); hypothesis of a post-COVID-19 IBS
Austhof et al. [45]	Population-based COVID-19 cohort	USA	1475 COVID-19 patients (N = 976 no digestive symptoms at baseline; N = 499 digestive symptoms)	Follow-up at 1.5, 3, 4.5, and 6 months Post-COVID-19 IBS diagnosed by Rome IV criteria	Average 6.2 months (175 days, S.D.: 61.6) IBS occurred in 3.0% (n = 15) of participants
Nazarewska et al. [46]	Prospective, single-center evaluation	Poland	257 COVID-19 patients	Follow-up at 3 and 6 months by Rome IV Diagnostic Questionnaire	After 3 and 6 months of follow-up IBS-like symptoms in 14 (5.4%) and IBS in 15 individuals (5.8%)
Farsi et al. [47]	Cross-sectional study	Iran	233 COVID-19 patients	Follow-up at 6 months by Rome IV criteria questionnaire	At 6 months 27 (11.6%) patients developed IBS
Siyal et al. [48]	Prospective	Pakistan	303 hospitalized COVID-19 patients without a prior history of IBS	Rome-IV criteria	IBS symptoms in 32 (10.6%) patients, of whom 17 (53.13%) diarrhea-predominant, 10 (31.25%) constipation-predominant, and 5 (15.62%) mixed-type IBS
Golla et al. [49]	Prospective follow-up cohort study	India	320 COVID-19 patients, 2 control groups (320 healthy spouses/family controls and 280 healthy COVID-19-negative controls)	Follow up at 1, 3, and 6 months by the Rome IV criteria	At 3 months, 8 (2.5%) had IBS-like symptoms
Zhang et al. [50]	Prospective	China	190 COVID-19 patients and 160 healthy controls	Follow-up for 1, 3, and 6 months by Rome III and Rome IV questionnaires	At 6 months 7 (3.7%) COVID-19 patients developed IBS

Abbreviations: IBS, irritable bowel syndrome; UD, uninvestigated dyspepsia.

**Table 3:** The main clinical trials on antiviral treatments in post-COVID-19 syndrome.

ClinicalTrials.gov ID	Phase	State	Official Title	Interventions/Treatments
NCT05668091 [74]	Phase 2	Completed	An Interventional Decentralized Phase 2, Randomised, Double-Blind, 2-Arm Study to Investigate the Efficacy and Safety of Orally Administered Nirmatrelvir/Ritonavir Compared with Placebo/Ritonavir in Participants with Long COVID	Drug: Nirmatrelvir Drug: Ritonavir Drug: Placebo
NCT06511063 [79]	Phase 2	Recruiting	Investigating the Feasibility of Repurposing HIV Antivirals in Adults with Long COVID	Truvada (tenofovir disoproxil/emtricitabine, TDF/FTC, Group 1) or Selzentry (Group 2), or a placebo (pill) (Group 3), taken daily for 90 days
NCT05595369 [80]	Phase 2	Completed	RECOVER-VITAL: A Platform Protocol for Evaluation of Interventions for Viral Persistence, Viral Reactivation, and Immune Dysregulation in Post-Acute Sequelae of SARS-CoV-2 Infection (PASC)	Experimental: Paxlovid 25-day dosing Experimental: Paxlovid 15-day dosing Placebo Comparator: Control
NCT05965726 [81]	Phase 2	Completed	RECOVER-VITAL: A Platform Protocol for Evaluation of Interventions for Viral Persistence, Viral Reactivation, and Immune Dysregulation in Post-Acute Sequelae of SARS-CoV-2 Infection (PASC)	Drug: Paxlovid 25-day dosing Drug: Paxlovid 15-day dosing Drug: Control
NCT06441955 [82]	Phase 4	Recruiting	COVID-19 Long Haul Syndrome: Undiagnosed Disorder Post COVID-19 Alternative Treatment Study.	Drug: Ritonavir-Boosted Nirmatrelvir (Paxlovid) Diagnostic Test: Physiological Evaluation Biological: Moderna COVID-19 Vaccine Behavioral: Biopsychological Behavioral: Behavioral (e.g., Psychotherapy, Lifestyle Counseling) Genetic: Genetic (including gene transfer, stem cell, and recombinant DNA) Combination Product: Multidisciplinary approach
NCT05576662 [83]	Phase 2	Completed	Selective Trial Of Paxlovid for PASC (STOP-PASC): Randomised Double-Blind Placebo-Controlled Pilot Trial of Paxlovid for the Treatment of PASC	Drug: Nirmatrelvir Drug: Placebo Drug: Ritonavir
NCT05823896 [84]	Phase 2	Completed	An Interventional, Double-Blinded, 2-Arm Study to Investigate the Efficacy of Orally Administered Nirmatrelvir/Ritonavir Compared with Placebo/Ritonavir in Non-hospitalized Adult Participants Suffering from Post-COVID	Drug: Nirmatrelvir/ritonavir Drug: Placebo/ritonavir
NCT05852873 [85]	Phase 3	Recruiting	PAxlovid loNg COVID-19 pRevention triAl With recruitMent In the Community in Norway	Drug: Nirmatrelvir/ritonavir Drug: Placebo
NCT05999435 [86]	Phase 2 Phase 3	Active, not recruiting	A Double-Blind, Randomised, Placebo-Controlled, Adaptive Phase 2/3 Study of the Efficacy of LAU-7b in the treatment of Adults with Long COVID and Moderate to Severe Symptoms	Drug: LAU-7b for 3 cycles Drug: LAU-7b for 1 cycle, then placebo Other: Placebo for 3 cycles
NCT06161688 [87]	Phase 2	Active, not recruiting	Placebo-Controlled, Randomised Trial of Ensitrelvir (S-217622) for Viral Persistence and Inflammation in People Experiencing Long COVID (PREVAIL-LC)	Drug: Ensitrelvir Other: Placebo
NCT04978259 [88]	Phase 4	Unknown	Long-term Follow-up of a Randomised Multicenter Trial on Impact of Long-COVID in Hospitalized COVID-19 Patients	Drug: Remdesivir
NCT05911906 [89]	Phase 4	Recruiting	An Open-label, Clinical Feasibility Study of the Efficacy of Remdesivir for Long-COVID.	Drug: Remdesivir
NCT06316843 [90]	Phase 2	Completed	A Randomised, Double-Blinded, Placebo-Controlled, Pilot Study of the Combination of Valacyclovir + Celecoxib (IMC-2) for the Treatment of Post-Acute Sequelae of SARS-CoV-2 Infection in Adults	Drug: Valacyclovir/celecoxib dose 1 Drug: Valacyclovir/celecoxib dose 2 Drug: Placebo
NCT06792214 [91]	Phase 4	Recruiting	Antiviral Strategies in the Prevention of Long-term Cardiovascular Outcomes Following COVID-19: The paxloviD/Remdesivir Effectiveness For the prEvention of loNg coviD (DEFEND) Clinical Trial	Drug: Nirmatrelvir/ritonavir Drug: Remdesivir

## Data Availability

All the data reviewed in this manuscript are available online, particularly in PubMed.
